# Effects of a coordinated program of psychomotricity and cognitive stimulation in vascular mild cognitive impairment and vascular dementia

**DOI:** 10.1590/1980-5764-DN-2025-0293

**Published:** 2025-11-14

**Authors:** Karla Valencia-Quiroz, Gerardo Maldonado-Paz, Adela Hernández-Galván

**Affiliations:** 1 Autonomous University of the State of Morelos, Center for Research in Cognitive Sciences, Cuernavaca Morelos, Mexico.; 2 Autonomous University of the State of Morelos, School of Higher Studies of Mazatepec, Miacatlán Branch Campus Morelos, Mexico.; 3 Autonomous University of the State of Morelos, Transdisciplinary Research Center in Psychology, Cuernavaca Morelos, Mexico.

**Keywords:** Cognitive Dysfunction, Dementia, Vascular, Psychomotor Performance, Cognitive Behavioural Therapy, Disfunção Cognitiva, Demência Vascular, Desempenho Psicomotor, Terapia Cognitivo-Comportamental

## Abstract

There is a growing interest in the study of vascular mild cognitive impairment (vMCI) and vascular dementia (VD) due to the increasing incidence of cardiovascular risk factors such as diabetes and overweight. The study of non-pharmacological interventions in individuals with vMCI and VD is crucial due to the cognitive and motor consequences of these conditions on the daily lives of individuals. Whereas most interventions are designed from a single disciplinary perspective, the intervention employed in this study integrated both psychomotor and cognitive approaches to concurrently stimulate participants’ embodied experience and cognitive processes.

**Objective:**

To assess the effects of an intervention combining cognitive stimulation and motor work on memory, attention, praxis, visuospatial abilities, and motor skills.

**Methods:**

A two-case study (one with vMCI and one with VD) with psychomotor and cognitive assessments pre- and post-intervention. The intervention combined psychomotor skills and cognitive stimulation and was applied over four months (32 sessions).

**Results:**

The participant with vMCI showed improvements in attention processes, executive functions, and psychomotor skills. The participant with VD showed improvements in memory and psychomotor skills. In both participants, stability was observed in the majority of the cognitive variables measured, along with improvements in processing visuospatial stimuli and neuropsychiatric symptoms (anxiety and apathy).

**Conclusion:**

Motor work can catalyze mental ability, promoting cognitive stimulation from the body to thought, which may be useful in improving and/or achieving stability in cognitive performance in individuals with vMCI and VD.

## RESUMO

Há um crescente interesse no estudo do comprometimento cognitivo leve vascular (CCLv) e da demência vascular (DV) em razão do aumento da incidência de fatores de risco cardiovascular, como diabetes e sobrepeso. O estudo de intervenções não farmacológicas em indivíduos com CCLv e DV é fundamental por conta das consequências cognitivas e motoras dessas patologias sobre a vida cotidiana das pessoas. Enquanto a maioria das intervenções é concebida de uma perspectiva disciplinar única, a intervenção utilizada neste estudo integrou abordagens psicomotoras e cognitivas com o objetivo de estimular simultaneamente a experiência incorporada e os processos cognitivos dos participantes.

**Objetivo:**

Avaliar os efeitos de uma intervenção que combina estimulação cognitiva e trabalho motor sobre processos de memória, atenção, praxias, habilidades visuoespaciais e motricidade.

**Métodos:**

Estudo de dois casos (um com CCLv e um com DV) com avaliação psicomotora e cognitiva antes e depois da intervenção. A intervenção combinou psicomotricidade e estimulação cognitiva, sendo aplicada durante quatro meses (32 sessões).

**Resultados:**

O participante com CCLv apresentou melhorias nos processos de atenção, funções executivas e psicomotricidade. A participante com DV apresentou melhorias em memória e psicomotricidade. Em ambos os participantes, observou-se estabilidade na maioria das variáveis cognitivas medidas, além de melhorias no processamento de estímulos visuoespaciais e nos sintomas neuropsiquiátricos (ansiedade e apatia).

**Conclusão:**

O trabalho motor pode funcionar como um catalisador para a habilidade mental, promovendo a estimulação cognitiva desde o corpo até o pensamento, o que pode ser útil para melhorar e/ou alcançar estabilidade no desempenho cognitivo de pessoas com CCLv e DV.

## INTRODUCTION

Mild cognitive impairment (MCI) refers to memory loss or cognitive function impairment beyond what is expected for an individual’s age without impacting daily living basic activities (BADLs)^1,2^. Vascular mild cognitive impairment (vMCI) is caused by vascular pathologies and can manifest in any MCI subtype: amnestic, multidomain, or non-amnestic single-domain or multidomain^3^.

Predominant cognitive impairments include difficulties in executive functions (EF), reduced processing speed, mental inflexibility, and information manipulation^4^. Depression, apathy, emotional lability, agitation, motor disturbances in gait and balance, and sleep disorders are frequently observed^5^.

Vascular dementia (VD) results from neuronal death caused by ischemic or hemorrhagic processes, cerebrovascular diseases, multi-infarcts, white matter lesions, and atrophy^3,6^. It is considered the second leading cause of dementia after Alzheimer’s disease (AD)^4,6,7^.

Cognitive impairments depend on the brain region affected by the vascular damage but generally include difficulties in EF, memory, attention, language problems, naming, and visuoconstructional skills^8,9^. Motor impairments include reduced gait speed, incoordination, apraxia, imbalance, falls, dysarthria, dysphagia, and rigidity^8^.

Hypertension, diabetes, and overweight are risk factors that increase the likelihood of developing vascular disease, vMCI, and VD. Identifying vMCI is crucial as it increases the probability of developing dementia^10^. In VD, shared risk factors and brain lesions also identified in AD may accelerate disease progression. These include parenchymal pathology, vascular pathology, loss of myelinated tissue, hippocampal lesions, amyloid deposits, and white matter lesions^4,6,7^.

The management of MCI and VD aims to slow cognitive decline through both pharmacological and non-pharmacological approaches, such as cognitive stimulation and physical exercise. However, these strategies are often implemented independently, as evidenced by several reviews and meta-analyses reporting studies that assess the effectiveness of cognitive stimulation in individuals with dementia in general^11,12^, in vMCI^13^, and VD^14^. These studies consistently report significant improvements in global cognitive measures such as the Mini-Mental State Examination (MMSE) or the Montreal Cognitive Assessment (MoCA), although findings regarding other outcomes—such as quality of life, depression, or behavioral symptoms—are more variable. A similar pattern is observed in systematic reviews and meta-analyses of clinical trials evaluating the impact of physical exercise interventions on cognition, which report improvements in this domain in individuals with dementia and general MCI^15,16^, as well as in those with vascular cognitive impairment, with positive effects on global cognition and EF^17^. However, other studies have failed to find a protective effect of physical exercise on vascular dementia^18^.

This is further supported by a scoping review^19^ that examined 91 non-pharmacological interventions in individuals with MCI and dementia. The review found that the most common types of interventions were standalone cognitive interventions (37 studies) and physical activity-based interventions (25 studies), while only four studies combined cognitive training with physical exercise. Although this review did not report on the effectiveness of the interventions, other studies did. For instance, a narrative review of 14 studies involving combined cognitive stimulation and physical exercise in individuals with MCI reported positive outcomes on global cognition, stress levels, and fatigue^20^. Similarly, a systematic review and meta-analysis of eight studies evaluating the efficacy of standalone cognitive interventions *versus* combined cognitive stimulation and physical exercise in individuals with mild cognitive impairment found that the combined intervention yielded greater improvements in global cognition than cognitive interventions alone^21^.

In recent years, there has been a growing trend toward proposing multidomain or multicomponent interventions, particularly as preventive strategies against cognitive decline. One notable example is the FINGERS study, in which individuals at high risk of cognitive impairment—primarily due to cardiovascular risk factors—participated in a comprehensive intervention including cognitive stimulation, physical exercise, medical monitoring, and nutritional intervention. In Finland, this approach succeeded in improving or maintaining cognitive function in the at-risk population^22^, and currently, the study is underway in 12 Latin American countries (LatAm-FINGERS)^23^. However, in such comprehensive interventions, it is challenging to discern the individual effects of each strategy or their possible combinations.

Therefore, it remains necessary and relevant to analyze the outcomes of multicomponent alternatives, particularly those that appear to yield the most promising results, such as the combination of cognitive interventions and physical exercise.

This study addresses this issue by proposing a psychomotor and cognitive intervention with transversal strategies applicable daily. This will contribute to developing more comprehensive theoretical frameworks that integrate knowledge from psychomotricity, motor cognition, and cognitive stimulation, aiming to propose interventions for both normal and pathological aging.

### Psychomotricity

Psychomotricity studies body movement, its effects on nervous system (NS) functioning, and its relationship to psychological development^24,25^. It is grounded in the study of the psychomotor system, which is embedded within the NS structures (brainstem, cerebellum, midbrain, and diencephalon) and is responsible for organizing motor functions such as muscle tone, balance, and laterality^26^.

Gerontopsychomotricity emerges from integrating gerontology and psychomotricity^27^ to enhance body and sensory awareness^28^, promoting independence and autonomy in older adults. Psychomotor techniques stimulate motor skills and cognition to support BADLs and independence, embodied life experiences that support adaptation to the inevitable physical changes associated with aging.

### Motor cognition

Motor cognition focuses on studying the representation of actions, from thought to action, using the simulation of real actions (motor imagery) to consciously generate an image of the activity^29^.

Motor imagery in rehabilitating patients with motor problems^30,31^ has been shown to promote brain modulation and cortical reorganization through neural plasticity. Additionally, it facilitates signal conductivity^32^.

It has been found that motor representations operate at different levels of action understanding: the kinematic level, the motor level, the goal level, and the level of intention, which involves understanding the reason for acting^33^. This explains why motor representations are crucial for imitation and tool use.

Apraxias are a consequence of the disruption of the mechanisms involved in action representation, being most evident in tool use (pragmatic meaning), including abstract forms of action (knowing what it is, how to use it, and what it is for)^29^. Various studies on individuals with apraxia have demonstrated difficulties performing pantomime actions, reflecting errors in movements’ kinematic and spatial trajectories, with disoriented and poorly coordinated movements spatially and temporally^34,35^. Other studies report difficulties in movement imitation, reflecting problems in selecting the different action components and explaining why difficulties are more pronounced in sequences of complex movements^36,37^.

### Cognitive stimulation

Cognitive stimulation is a multisensory intervention aimed at improving cognition and social functioning through activities that consistently stimulate cognitive processes (memory, language, attention, EF, and others) to slow cognitive decline and create new pathways of brain functioning through experience and learning^38,39^.

The multisensory and integrative approach is grounded in the three fundamental types of neuroplasticity (synaptic plasticity, neurogenesis, and functional plasticity). It aims to improve cognitive functioning, reduce dependency in older adults, and enhance their remaining cognitive abilities and skills^40^.

Cognitive stimulation programs for individuals with MCI report significant improvements in cognitive performance (reality orientation, spatial and temporal orientation, memory, language, calculation, praxis, gnosis, and EF) and mood disorders (anxiety and depression)^41^. Current evidence suggests that stimulation should encompass sensory, motor, and cognitive aspects to stimulate multiple neural networks, facilitating brain plasticity processes^40,42,43^.

Given the above, this research aimed to evaluate the effects of an intervention combining cognitive and motor stimulation on memory, attention, praxis, and visuospatial processes in one person with vMCI and another with early-stage VD, using mental imagery processes, psychomotricity, and cognitive exercises as support.

## METHODS

A two-case study with a quantitative approach and pre-experimental design, including pre- and post-intervention assessments.

Inclusion criteria: individuals diagnosed with MCI or dementia of probable vascular etiology in the mild to moderate stages (2 to 4 on the Reisberg scale), confirmed by imaging studies, neuropsychological tests, and the opinion of a psychogeriatrician with high specialization in dementia, without severe visual or motor difficulties. Due to the specific criteria and in order to have better control of them, it was decided to carry out the selection of participants within the Memory Clinic of the Transdisciplinary Research Center in Psychology.

### Participants

Participant 1 (P1): Male, 80 years old, with a high level of education (16 years). Medical history includes hypertension and anxiety. Independent in performing BADLs and instrumental activities, moderate physical activity (daily walks). Diagnosed with amnestic MCI of a single domain with vascular etiology (small vessel disease, Fazekas stage 2), with approximately two years of progression. Adheres to pharmacological treatment for underlying conditions, which includes galantamine.

Participant 2 (P2): Female, 77 years old, with a high level of education (12 years). Medical history includes hypertension, cerebral microinfarcts (mainly in the right hippocampus), and smoking. Independent in performing BADLs; dependent for most instrumental activities. Diagnosed with mild major neurocognitive disorder of vascular etiology due to cerebral microinfarcts, with approximately four years of progression. Presence of apathy as the only neuropsychiatric symptom. Pharmacological treatment for hypertension, without prescription of cognitively active medications.

### Materials and instruments

The assessment of participants was conducted based on a clinical interview that included a structured anamnesis, a cognitive screening test (MMSE), and a comprehensive battery (NEUROPSI Attention and Memory) designed to thoroughly evaluate key cognitive domains of interest, namely attention, memory, and executive functioning. In addition, the Global Deterioration Scale was employed to estimate the degree of cognitive impairment, and the Psychomotor Battery was used to assess psychomotor skills. The validated version of the MMSE for the Mexican population was used, and the NEUROPSI Attention and Memory battery was specifically developed and validated in a Mexican population.

All instruments (with the exception of the anamnesis) were administered both before and after the intervention. A brief description of each is provided below.

- Brief anamnesis: a structured questionnaire that investigates medical history, current health status, medications, habits, and lifestyle;
- MMSE^44^: detects the presence of cognitive impairment, evaluating temporal and spatial orientation, immediate and delayed memory, concentration and calculation, language, and visuoconstructive praxis. It has been validated in the Mexican population. A Receiver Operating Characteristics (ROC) curve analysis indicated that a cutoff score of 23/30 is optimal for the Mexican population, yielding a sensitivity of 0.97, a specificity of 0.88, and a reliability coefficient with a Cronbach’s alpha of 0.89;
- NEUROPSI Attention and Memory^45^: 29 subtests that evaluate attention processes (selective, sustained, and attentional control) and memory (working memory, short-term memory, long-term verbal memory, and visuospatial memory). Raw scores from the subtests are converted into standardized scores with a mean of 10 and a standard deviation of 3. Additionally, it is possible to obtain composite standardized scores for the attention and executive function subtests, for the memory subtests, and for the combined attention and memory subtests. The battery provides normative profiles for nine age groups ranging from six to 85 years and across three levels of educational attainment. The test was designed and validated in the Mexican population;
- Global Deterioration Scale^46^: evaluates the progression of impairment in terms of severity and classifies cognitive decline into seven stages;
- Psychomotor Battery (PMB)^26^: evaluates tone, balance, laterality, body awareness, spatial-temporal structuring, global praxis, and fine praxis, providing a psychomotor profile.

The intervention was conducted in a spacious, clear, and easily accessible area. The psychomotor materials included hoops, cones, balls, colored ribbons, fine psychomotor materials, and wooden keys. For cognitive stimulation, exercises from specialized books for older adults with cognitive impairment or dementia^47,48,49,50,51,52^, logical blocks, color towers, and cards with various stimuli were employed.

### Procedure

The process began with the reading and signing of the informed consent form. Subsequently, neuropsychological and psychomotor tests were administered in three individual sessions, each lasting 60 minutes. The intervention was carried out over four months, after which the participants were re-evaluated using the aforementioned instruments.

### Intervention

Based on the deficits identified in both conditions, an intervention was designed in four modules, each lasting one month. Two sessions per week were conducted for four months (32 sessions in total). The psychomotor intervention focused on body awareness, postural control, multisensory perception, balance, joint stability, proprioception, the representation of bodily movements through motor imagery, as well as fine and gross motor coordination. The cognitive processes addressed included: motor control, visual and auditory perception, motor representation, visual representation, visuospatial skills, inhibitory control, attention, praxis, and memory.

The intervention was individually applied. Each session was divided into two parts: the first half hour focused on psychomotor work and the second half on cognitive stimulation. A five-minute break between the psychomotor and cognitive stimulation segments was provided for physical recovery, reflection on the work completed, and to introduce the cognitive stimulation tasks. At the end of each session, transfer and metacognitive strategies to daily life were emphasized by discussing the everyday activities where participants could apply what they had learned during the session. In all sessions, the physical and emotional state of the participants was monitored, paying attention to their frustration tolerance, motivation for the tasks, and physical condition to avoid exhaustion and muscle fatigue.

### Data analysis

The modified Crawford *t*-test was employed to compare two independent cases^53^, allowing for one-tailed and two-tailed significance testing, with point estimates and confidence intervals. In case studies, these comparisons enable testing for double dissociation, meaning comparing the performance of a participant across tasks^54^.

The modified Crawford *t*-test was used to compare the scores obtained by each participant on the neuropsychological tests before and after the intervention. The software used was the classical method Compare Two Cases (C_CTC.exe)^53^.

### Ethical considerations

The Research Ethics Committee of the Transdisciplinary Research Center in Psychology (CITPsi) approved the research protocol, registered under number 101122-91. This committee is certified by the National Bioethics Commission of the Ministry of Health of Mexico. A Privacy Notice was also signed.

## RESULTS

P1, diagnosed with vMCI, obtained a Normal score (29) on the MMSE in both the pre-test and post-test. In the NEUROPSI, improvements were observed in all three total scores calculated in the test, as well as a positive change in the level of impairment in attention and EF, moving from Mild-Moderate Impairment to Normal (Table 1). The subtests in the ranges of Mild-Moderate Impairment and Severe Impairment in the pre-test showed positive changes in the normalized score of the post-test; likewise, changes were observed in the normalized scores of other subtests (Table 2).

**Table 1.** Total scores of the pre-test and post-test of the NEUROPSI for the participant with vascular mild cognitive impairment.

Total Scores	Pre-test			Post-test		
	Raw score	Normalized score	Level of impairment	Raw score	Normalized score	Level of impairment
Total Attention and Executive Functions*	77	78	Mild to Moderate Impairment	85	92	Normal
Total Memory	108	93	Normal	114	96	Normal
Total Attention and Memory	185	89	Normal	199	95	Normal

**Table 2.** Normalized scores and p-values of the NEUROPSI test for the participant with vascular mild cognitive impairment.

	Subtest	Normalized score Pre-test	Normalized score Post-test	Modified Crowford *t*	Two-tailed p-value	Confidence interval
Orientation	Orientation to time	10	10	0.000	1.000	
	Orientation to space	10	10	0.000	1.000	
	Orientation to person	10	10	0.000	1.000	
Attention and Concentration	Digits Span in Progression	13	13	0.000	1.000	
	Progressive Cubes^	6	9	-2.121	0.045*	-2.75 to -1.48
	Visual Detection^	4	7	-0.408	0.687	-0.53 to -0.28
	Digit Detection	11	11	0.000	1.000	
	Successive Sequences	12	13	-0.505	0.618	-0.65 to -0.35
Working Memory	Digit Span in Regression	11	11	0.000	1.000	
	Regression Cubes	8	8	0.000	1.000	
Memory (Encoding)	Spontaneous Memory Curve	8	8	0.000	1.000	
	Paired Associates	9	10	-0.244	0.809	-0.31 to -0.17
	Logical Memory^	4	8	-1.179	0.251	-1.53 to -0.82
	Rey-Osterrieth Figure	8	8	0.000	1.000	
	Faces	12	12	0.000	1.000	
Memory (Recall)	Spontaneous Verbal Memory	7	7	0.000	1.000	
	Verbal Memory with Cues	7	8	-0.221	0.827	-0.28 to -0.15
	Verbal Memory by Recognition	7	7	0.000	1.000	
	Paired Associates	10	8	0.505	0.618	0.35–0.65
	Logical Memory	9	10	-0.321	0.751	-0.41 to -0.22
	Rey-Osterrieth Figure	13	12	0.088	0.930	0.06–0.11
	Face Recognition	8	11	-2.121	0.045*	-2.75 to -1.48
Executive Functions	Category Formation	12	9	0.493	0.626	0.3–0.6
	Semantic Verbal Fluency	9	9	0.000	1.000	
	Phonological Verbal Fluency	19	8	3.111	0.005*	2.17–4.04
	Non-verbal Fluency^	4	16	-3.536	0.001*	-4.59 to -2.47
	Motor Functions	7	11	-0.786	0.440	-1.02 to -0.55
	Stroop Interference Time	3	1	0.211	0.834	0.14–0.27
	Stroop Interference Accuracy	11	13	-0.393	0.698	-0.51 to -0.27

Notes: ^the tests that obtained scores ranging from Mild to Moderate Impairment and Severe Impairment in the pre-test

*p<0.05; in the NEUROPSI, normalized scores of 6 or lower are considered abnormal or within the impairment range.

The results from the specific subtests show that in 13 of the 29 subtests of the NEUROPSI, the scores remained the same between the pre-test and post-test evaluations, while 11 showed improved scores in the post-test. Significant changes were observed in three subtests, and clinically significant changes were found in four subtests. Clinical significance is described as “performance that was initially abnormal and falls within the normal range after treatment^55^ (p. 914). In five subtests, the scores were lower in the post-test, with a significant negative change recorded in phonological fluency, although the normalized scores obtained in both the pre-test and post-test for all subtests, except for one (Stroop time), fall within the normal range.

**Figure 1.**
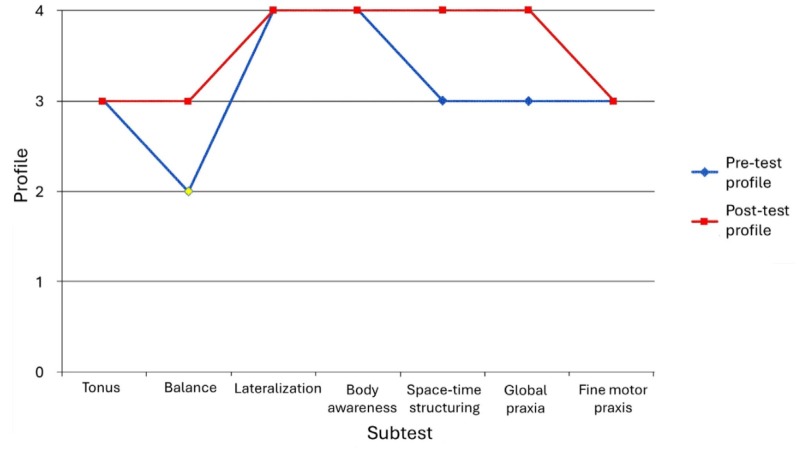
Pre-test and post-test scores on the Psychomotor Battery of the participant with vascular mild cognitive impairment.

Note: Level 1 is an apraxic profile (weak, uncoordinated). Level 2 is a dyspraxic profile (difficult control). Level 3 is a eupraxic profile (good, adequate). Level 4 is a hyperpraxic profile (excellent, controlled).

In the PMB, positive changes were observed in three subtests, while the scores of the remaining four subtests remained stable (Figure 1). The changes occurred in gross motor skills, such as balance and global praxis. The participant maintained a profile between the Eupractic and Hyperpractic levels.

P2, diagnosed with VD, obtained a normal score (24) on the MMSE in the pretest and post-test. In the NEUROPSI, a positive change was observed in the level of impairment in the memory area (total score), moving from Severe Impairment to Mild-Moderate Impairment (Table 3).

**Table 3.** Total scores of the pre-test and post-test of the NEUROPSI for the participant with vascular dementia.

Total Scores	Pre-test			Post-test		
	Raw Score	Normalized Score	Level of Impairment	Raw Score	Normalized Scored	Level of Impairment
Total Attention and Executive Functions	64	54	Severe Impairment	65	56	Severe Impairment
Total Memory	57	64	Severe Impairment	67	70	Mild to Moderate Impairment
Total Attention and Memory	121	61	Severe Impairment	132	65	Severe Impairment

Participant P2 obtained the same scores in the pre- and post-intervention evaluations in 13 of the 29 subtests of the NEUROPSI. In ten subtests, the scores were improved, and a significant improvement was observed in one test. Clinical significance was observed in five subtests, with scores shifting from the Impaired to Normal range. However, in six subtests, the post-test scores were worse, and three subtests showed significantly worse scores in the post-intervention evaluation, although the latter subtest remained within the normal range (Table 4).

**Table 4.** Normalized scores and p-values of the participant with vascular dementia.

	Subtest	Normalized Score Pre-test	Normalized Score Post-test	Modified Crowford t	Two-tailed p-value	Confidence Interval
Orientation	Orientation to time	1	1	0.000	1.000	
	Orientation to space	10	10	0.000	1.000	
	Orientation to person	10	10	0.000	1.000	
Attention and Concentration	Digits Span in Progression	13	9	4.041	0.0005*	2.82–5.25
	Progressive Cubes^	6	9	-2.121	0.045*	-2.75 to -1.48
	Visual Detection^	4	3	-0.136	0.893	0.09–0.17
	Digit Detection	8	11	-1.928	0.067	-2.50 to -1.34
	Successive Sequences	12	12	0.000	1.000	
Working Memory	Digit Span in Progression	11	11	0.000	1.000	
	Regression Cubes^	5	8	-2.357	0.028*	-3.06 to -1.64
Memory (Encoding)	Spontaneous Memory Curve	7	7	0.000	1.000	
	Paired Associates^	6	8	-0.488	0.630	-0.63 to -0.34
	Logical Memory	7	7	0.000	1.000	
	Rey-Osterrieth Figure^	1	1	0.000	1.000	
	Faces	7	7	0.000	1.000	
Memory (Recall)	Spontaneous Verbal Memory	7	7	0.000	1.000	
	Verbal Memory with Cues^	5	6	-0.221	0.827	-0.28 to -0.15
	Verbal Memory by Recognition	7	9	-0.337	0.739	-0.43 to -0.23
	Paired Associates^	5	10	-1.263	0.220	-1.64 to -0.88
	Logical Memory^	3	1	0.643	0.527	0.45–0.83
	Rey-Osterrieth Figure^	4	4	0.000	1.000	
	Face Recognition	11	8	2.121	0.045*	1.48–2.75
Executive Functions	Category Formation^	3	5	-0.329	0.745	-0.42 to -0.23
	Semantic Verbal Fluency^	5	8	-0.386	0.703	-0.50 to -0.27
	Phonological Verbal Fluency	9	1	2.263	0.034*	1.58–2.94
	Non-Verbal Fluency	9	11	-0.589	0.561	-0.76 to -0.41
	Motor Functions	10	10	0.000	1.000	
	Stroop Interference Time^	1	1	0.000	1.000	
	Stroop Interference Accuracy	11	9	0.393	0.698	0.27–0.51

Notes: ^the tests that obtained scores ranging from Mild to Moderate Impairment and Severe Impairment in the pre-test

*p<0.05; in the NEUROPSI, normalized scores of 6 or lower are considered abnormal or within the impairment range.

**Figure 2.**
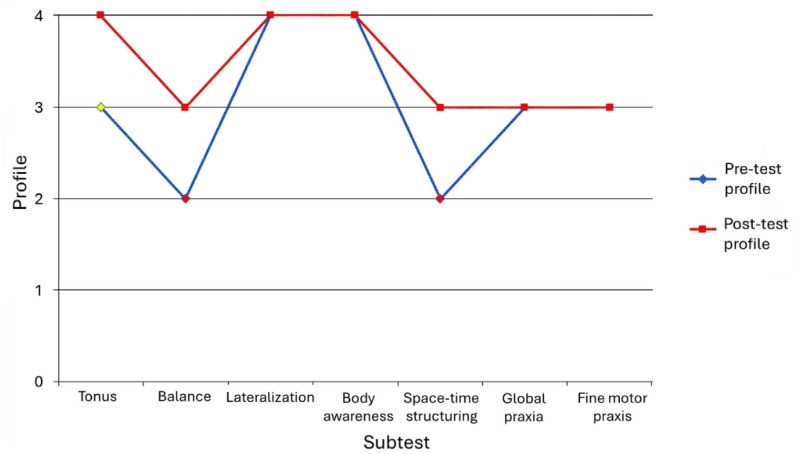
Pre-test and post-test scores on the Psychomotor Battery of the participant with vascular dementia.

Note. Level 1 is an apraxic profile (weak, uncoordinated). Level 2 is a dyspraxic profile (difficult control). Level 3 is a eupraxic profile (good, adequate). Level 4 is a hyperpraxic profile (excellent, controlled).

In the PMB, positive changes were observed in three subtests, while the scores of the remaining four subtests remained stable (Figure 2). The changes were observed in motor control, tone, and balance. The participant maintained an Eupractic and Hyperpractic level.

## DISCUSSION

According to the study’s objectives, the results indicate that the intervention had positive effects on memory, attention, EF, and motor skills in P1; and on memory, EF, and motor skills in P2. These results show similarities with previous research on the effects of psychomotor exercise on cognitive processes^56,57^, reporting improvements in cognitive functions (attention, EF, orientation, short-term memory, and constructive praxis) and in motor activities.

Both participants showed improvement in tasks involving immediate memory and executive processes with visual and visuospatial stimuli (cubes in progression, cubes in regression, and non-verbal fluency). This improvement can be attributed to the work with visuomotor and visuospatial coordination exercises as part of the psychomotor stimulation. The results in the psychomotor aspect confirm that psychomotricity in older adults can have a preventive and preservative function for tone, postural control, body image, and spatial-temporal organization^58^.

The work involving motor imagery is strengthened when combined with cognitive stimulation targeting various types of praxis (ideomotor, ideational, visuoconstructive, facial, and dressing). This integration is facilitated through the transfer of strategies that foster interrelations among these functions. Motor imagery, in conjunction with the stimulation of different forms of praxis, may contribute to the activation of the cerebellum—a structure of significant importance due to its involvement in both motor and cognitive functions, including executive functioning, memory, language, and visuospatial abilities^59^. Furthermore, interventions focused on visual representation, visual perception, and spatiotemporal orientation complement the necessary work to maintain a stable praxis performance.

Based on the aforementioned considerations and the results obtained, it is possible to affirm that the use of motor imagery can support the enhancement of executive functions, particularly by emphasizing the planning of sequences and resources, as well as the monitoring of described actions and the evaluation of performance at the moment the exercise is mentally enacted with clarity and precision.

These results align with slowing cognitive decline through non-pharmacological interventions^60^, in this case combining cognitive stimulation and psychomotricity. Evidence of this is that both participants obtained the same score in the pre-test and post-test on the MMSE. Both showed improvements in the three total scores of the NEUROPSI and maintained the same scores or exhibited some improvement in 80% of the NEUROPSI subtests.

In patients with MCI who progress to dementia, a mean annual decrease of 1.3 points on the MMSE is estimated^61^, and 1.4 points per year in patients with AD^62^. Another study^63^ reports that newly diagnosed dementia patients lose 1.5 points on the MMSE in the first year, and, by the third year of diagnosis, they lose more than 2 points per year due to the progression of cognitive decline. Although our study spanned four months between the pre-test and post-test evaluations, the stability found in the MMSE scores for both participants could reflect a slowing of cognitive decline due to the intervention. This is especially true for P2, where the decline would likely have been more pronounced since no cognitive-enhancing medications were prescribed and the disease had been evolving for over three years.

The positive effect of psychomotor stimulation was also observed in P1, who, in the pre-test, showed difficulties in global praxis on the PMB, specifically in the imitation of movements and in the sequencing of complex movements^37,38^. These difficulties improved in the post-test, along with the motor functions measured in the NEUROPSI, which assesses executive control over movement. This improvement is attributed to the work on motor imagery, which emphasized the representation of an action, the evocation of bodily sensations, and the planning and description of movements.

Verbal fluency (VF) has been identified as one of the most significant signs for the diagnosis of dementia^64^, as well as a predictor for the progression from MCI to dementia^65^. This task has a strong executive component, requiring a volitional and active process of selecting specific items, inhibiting unrelated elements, and working memory to avoid repetition of already evoked items^64^. Semantic fluency (SF) and phonological fluency (PF) rely on different brain structures. While SF requires declarative memory, specifically in access to lexical-semantic networks that rely on extraperisylvian and medial temporal areas, PF requires a search strategy since words beginning with a letter are not organized in categories that can be accessed, requiring the involvement of the prefrontal cortex^64^. Therefore, PF is more difficult than SF^66^, and SF is more vulnerable to the aging process^67,68^, declining more rapidly than PF in healthy individuals^69^ due to the mnemonic component involved in this task.

Individuals with cognitive impairment produce fewer words in SF and PF than those with healthy aging^70^. In SF, healthy individuals typically evoke between 18 and 22 words^65^, individuals with MCI average 12 words, and those with VD average eight words. In the present study, P1 with vMCI showed a normal performance in the pre-test and post-test (18 and 17 words, respectively), falling within the 50^th^ percentile in both evaluations according to Mexican norms^71^. Meanwhile, P2 with VD improved from the 5^th^ percentile in the pre-test to the 25^th^ percentile in the post-test. These findings suggest stability and improvement in this task for both participants after the intervention, likely linked to memory and executive processes improvements.

Regarding PF, individuals with MCI typically produce 37 words, and those with dementia 20 words^69^. Both participants in the present study had lower results in PF in the post-test (P1 evoked 12 words and P2, six words), which aligns with the greater impairment of executive functions in vMCI. This suggests that the intervention did not positively influence this task. However, it is essential to highlight that both participants improved in non-verbal fluency in the post-test, which also requires executive processes but with visuospatial stimuli. This process was specifically targeted during the psychomotor intervention.

The main neuropsychiatric characteristics of vMCI and VD include anxiety, depression, and apathy^8,72^. During the sessions, particularly in the final phase involving transfer and metacognitive exercises, P1 showed a marked reduction in anxiety episodes and was able to identify and verbalize some of their triggers. In turn, P2 exhibited decreased apathy, which enabled her to engage more actively in applying the strategies at home with her family. It is recommended that future replications of this study incorporate instruments to assess anxiety and depression in participants before and after the intervention, in order to verify the potential impact of this approach on these psychological dimensions.

It is important to highlight that adherence to the program was a critical factor in achieving the observed outcomes, as both participants completed all scheduled sessions. The following elements were deemed essential in promoting this adherence:

- the participants’ intrinsic motivation to engage in the intervention activities;
- their personal commitment to implementing the strategies in daily life contexts;
- the support provided by their environment, which facilitated opportunities for practice and acceptance.

In conclusion, the results suggest that an intervention combining psychomotor therapy and cognitive stimulation positively affects cognitive processes. Motor work may contribute to mental ability, meaning that cognitive stimulation, from the body to thought, can be helpful to improve and/or stabilize cognitive performance in individuals with vMCI and VD.

The main contributions of this study are the use of strategies that promote awareness of the relationship between the body and thought, as well as between the skills worked on in a sensorimotor mode and an abstract manner. The transfer of cognitive abilities to BADLs through language and the use of strategies to resolve situations in various environments where the individual functions are also emphasized. Regarding the intervention, it is recommended to maintain a frequency of two to three times per week, considering the individuals’ physical conditions and avoiding fatigue or stress from work overload.

The results are limited due to the small number of cases included and the duration of the intervention. Therefore, it is suggested that future interventions consider increasing the sample size and extending the intervention period beyond four months.

## Data Availability

The datasets generated and/or analyzed during the current study are available from the corresponding author upon reasonable request.
